# A novel 14q13.1–21.1 deletion identified by CNV-Seq in a patient with brain-lung-thyroid syndrome, tooth agenesis and immunodeficiency

**DOI:** 10.1186/s13039-019-0463-z

**Published:** 2019-12-19

**Authors:** Xuyun Hu, Jun Liu, Ruolan Guo, Jun Guo, Zhipeng Zhao, Wei Li, Baoping Xu, Chanjuan Hao

**Affiliations:** 1 Beijing Key Laboratory for Genetics of Birth Defects, Beijing Pediatric Research Institute; MOE Key Laboratory of Major Diseases in Children; Genetics and Birth Defects Control Center, Beijing Children’s Hospital, Capital Medical University, National Center for Children’s Health, Beijing, 100045 China; 2 China National Clinical Research Center of Respiratory Diseases, Respiratory Department of Beijing Children’s Hospital, Capital Medical University, National Center for Children’s Health, Beijing, 100045 China

**Keywords:** 14q13 deletion, CNV-seq, Brain-lung-thyroid syndrome, Immunodeficiency

## Abstract

**Background:**

Chromosome 14q11-q22 deletion syndrome (OMIM 613457) is a rare genomic disorder. The phenotype heterogeneity depends on the deletion size, breakpoints and genes deleted. Critical genes like *FOXG1*, *NKX2*–1, *PAX9* were identified.

**Case presentation:**

We performed whole exome sequencing (WES) and copy number variation sequencing (CNV-seq) for a patient with mild speech and motor developmental delay, short stature, recurrent pulmonary infections, tooth agenesis and triad of brain-lung-thyroid syndrome. By using CNV-seq, we identified a 3.1 Mb de novo interstitial deletion of the 14q13.2q21.1 region encompassing 17 OMIM genes including *NKX2–1*, *PAX9* and *NFKBIA*. Our patient’s phenotype is consistent with other published 14q13 deletion patients.

**Conclusion:**

Our results showed the combination of WES and CNV-seq is an effective diagnostic strategy for patients with genetic or genomic disorders. After reviewing published patients, we also proposed a new critical region for 14q13 deletion syndrome with is a more benign disorder compared to 14q11-q22 deletion syndrome.

## Background

Chromosome 14q11-q22 deletion syndrome (OMIM 613457) is a genomic disorder characterized by microcephaly, dysmorphic facies, psychomotor delay and failure to thrive. The associated phenotype is heterogeneous, depending on the size and variable breakpoints [1–3]. Some major features can be explained by haploinsufficiency of critical genes like *FOXG1*, *NKX2*–1, *PAX9*, etc. [4, 5]. Patients with interstitial deletions involving 14q13.1q21.1 are rarely reported and all these patients had similar features. *NKX2*–1 and *PAX9* in 14q13 are considered to be the critical genes causing brain-lung-thyroid syndrome (BLTS, OMIM 610978) and tooth agenesis (OMIM 604625) features, respectively [6, 7]. More recently, *NFKBIA* was purposed to be responsible for immunodeficiency these patients [8].

In our study, we report a new patient with 14q13.1q21.1 distal microdeletion syndrome. Copy number variation sequencing (CNV-seq) revealed a de novo 3.10 Mb sized deletion (chr14: 35,268,524–38,367,321). We detailed described this patient’s phenotype and reviewed all reported patients with similar breakpoints encompassing *NFKBIA*, *NKX2*–1 and *PAX9*, and provided more information of relationships between clinical features and deleted genes.

## Case presentation

The patient is a 15-year-old female born to nonconsanguineous parents by nature labor after full-term gestation. Family history is unremarkable. She was diagnosed as aspiration pneumonia at birth and stayed in hospital for 4 days. Developmental delay was noticed after birth. She could not sit until 12 months. She could speak after 24 months and walk after 27 months. Dysarthria and ataxia were also unnoticed since after. At the age of 2 years, she went to hospital for speech and motor developmental delay. The symptoms were relieved after rehabilitation training. She was also diagnosed as short stature when she was 14 and received growth hormone therapy for 9 months. Her height increased from 132 cm (− 4.7 SD) to 142 cm (− 3.2 SD), resulting in an annual growth velocity of 13 cm.

**Fig. 1:**
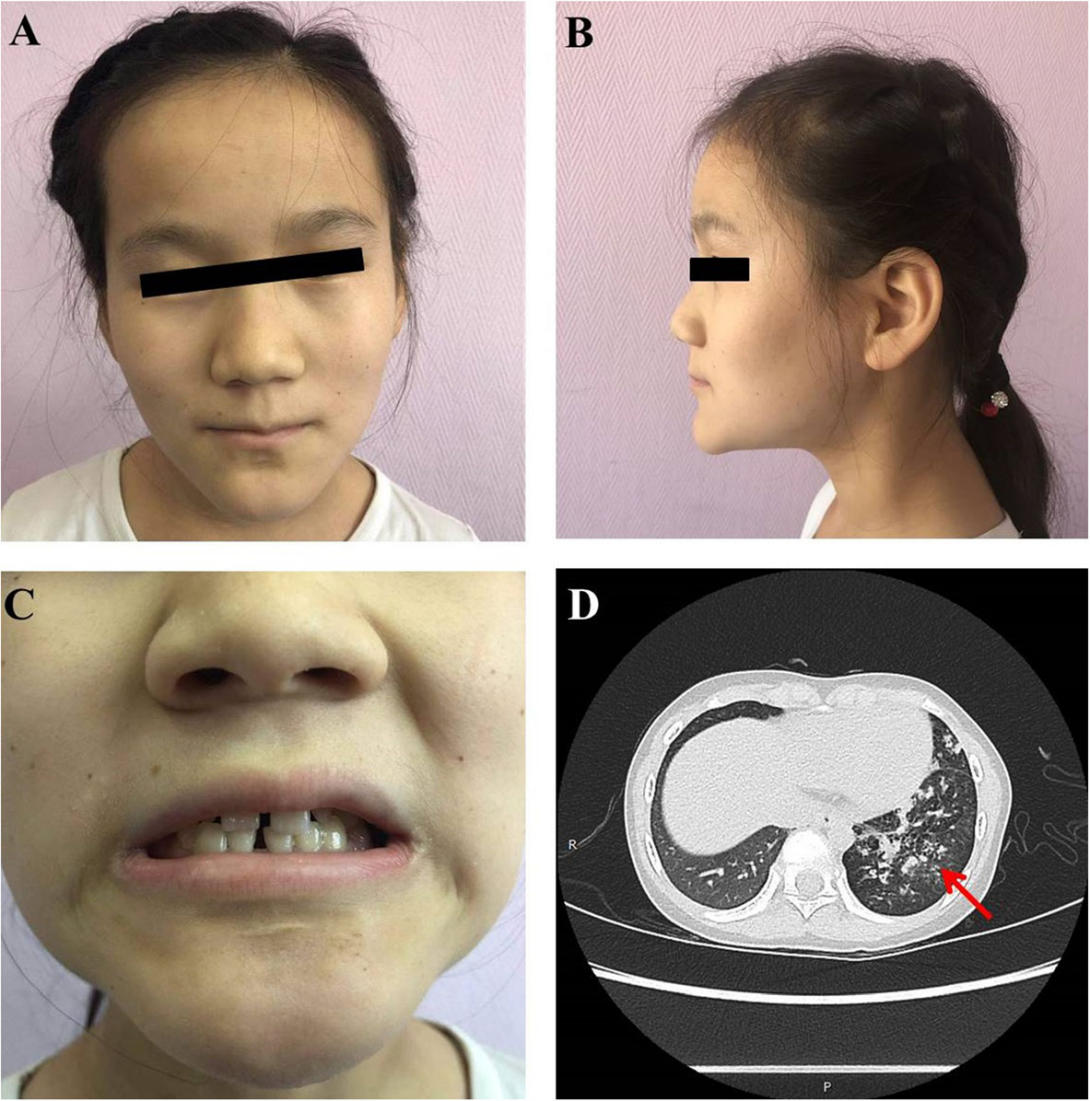
Phenotype of the patient. **a**-**b** This patient did not have characteristic facial feathers of 14q11-q22 deletion syndrome. **c** The patient had normal primary dentition and agenesis of permanent teeth. **d** Thoracic CT showed interstitial and parenchymal lesions and bronchiectasis in both lungs (red arrow)

At the age of 15 years, she was referred to the Department of Respiration, Beijing Children’s Hospital for continuous cough and expectoration for 3 years. Her height and weight were below 3rd percentile. She did not have obvious facial anomalies but hypohidrosis and tooth agenesis (missing secondary dentition including molars and premolars) were noticed (Fig. 1a-c). She also had punctate pigmentation was noticed on her back. Mental development was delayed compared to peer children. Immune system examination revealed decreased IgG (3.69 g/L), other immunoglobulins and lymphocyte subsets were normal. Paranasal sinusitis and anemia were diagnosed. She had increased thyroid-stimulating hormone (TSH, 11.983 mIU/L), deceased triiodothyronine (T3, 58.79 ng/dL) and normal tetraiodothyronine (T4, 4.81 μg/dL), indicating a compensated hypothyroidism. She also had delayed development of the secondary sexual characters. Till this visit, she did not have menarche when she was 15. Her bone age was also delayed (BA = 10).

Thoracic computed tomography (CT) revealed interstitial and parenchymal lesions and bronchiectasis in both lungs, dominated with interstitial lesions, and mucus plugs was found in right upper lobe and left lower lobe (Fig. 1d). Ultrasonography of knee joint showed very limited amount of effusion in bilateral suprapatellar capsule. Routine blood test, coagulation function, and screening for infectious diseases, abdominal ultrasonography, ultrasonic cardiography, cranial CT and paranasal sinus CT were all normal.

**Fig. 2:**
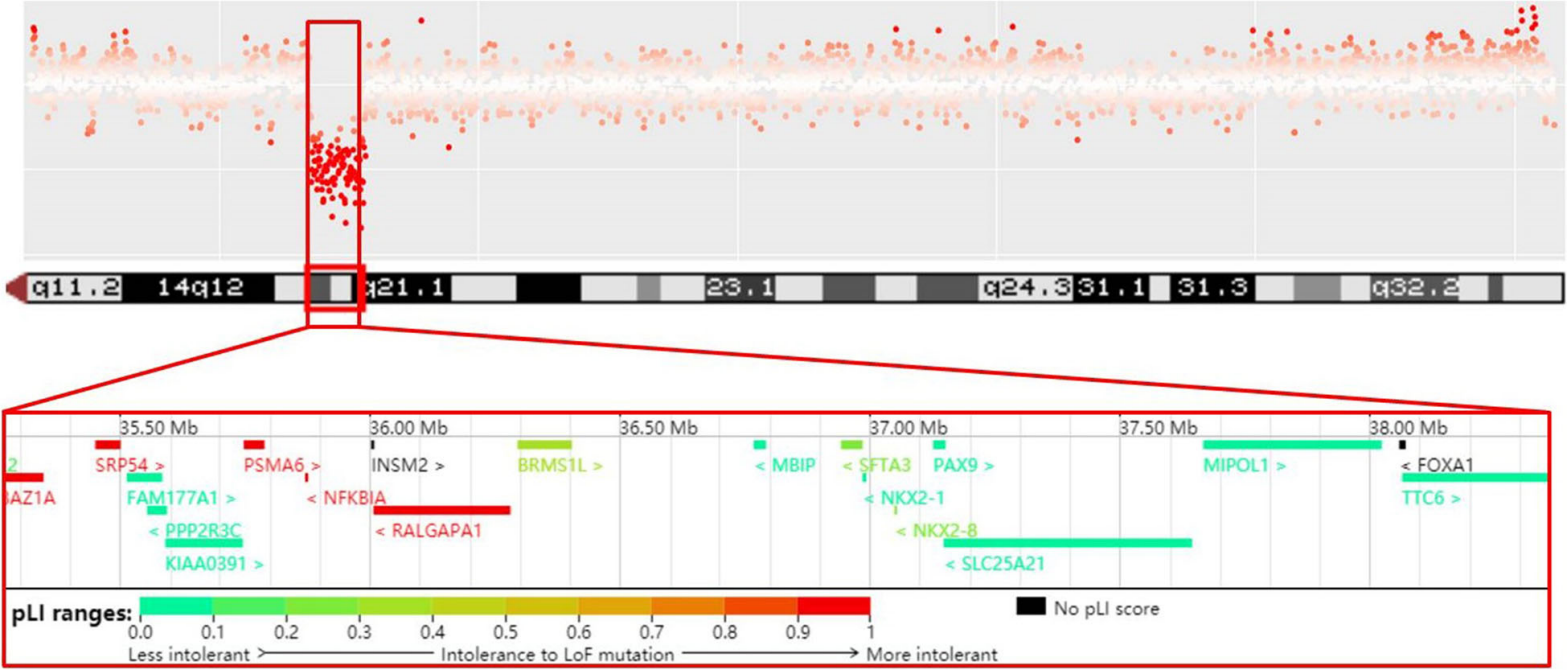
A de novo 3.1 Mb deletion on 14q13.1q21.1 was identified in the patient. The deletion encompasses 17 OMIM genes and the pLI (probability of LoF intolerant) value of each gene is shown in colors. The deletion shown is detected by CNV-seq

DNA was isolated from peripheral blood samples obtained from the proband and her parents by using Gentra Puregene Blood Kit (QIAGEN, Hilden, Germany). 200 ng genomic DNA of each individual was sheared by Biorupter (Diagenode, Belgium) to acquire 150~200 bp fragments. The ends of DNA fragment were repaired and Illumina Adaptor was added (Fast Library Prep Kit, iGeneTech, Beijing, China). After sequencing library were constructed, the whole exome was captured with AIExome Enrichment Kit V1 (iGeneTech, Beijing, China) and sequenced on Illumina NovaSeq 6000 (Illumina, San Diego, CA) with with 150 base paired-end reads. Raw reads were filtered to remove low quality reads by using FastQC. Clean reads were mapped to the reference genome GRCh37. Single nucleotide variants (SNVs) were annotated and filtered by TGex (http://app.genecards.cn) and classified following the American College of Medical Genetics and Genomics and the Association for Molecular Pathology interpretation standards and guidelines [9]. To identify large copy number variations (CNVs), part of the sequencing library was sequenced directly on Illumina NovaSeq 6000 and each sample yielded one Gigabase Raw data. An in-house pipeline was applied to map and call CNVs and the health parents were used as control samples [10]. Clean reads were mapped to the reference genome GRCh37. Database of Genomic Variants, DECIPHER database, ClinVar, OMIM and ClinGen were used for interpretation and classification of the clinical significance of candidate CNVs according to previously reported guidelines [11]. By whole exome sequencing (WES), 30,636 SNVs and small indels were called from 14,549.59 Mb Clean bases (Target mean depth was 122.23X) in the proband. After analysis and interpretation, none of these variants could explain the proband’s phenotype. However, CNV-seq revealed a de novo 3.1 Mb deletion on 14q13.1q21.1 (chr14: 35,268,524–38,367,321) (Fig. 2). The deletion encompasses 17 OMIM genes including *BAZ1A*, *SRP54*, *PPP2R3C*, *KIAA0391*, *PSMA6*, *NFKBIA*, *INSM2*, *RALGAPA1*, *PTCSC3*, *MBIP*, *SFTA3*, *NKX2–1*, *NKX2–8*, *PAX9*, *SLC25A21*, *MIPOL1* and *FOXA1*. The deletion was not present in the parents. WES reads depth data was used to validate this deletion (log2 = − 1.00428, mean depth = 50.02X, probes number = 379). Informed consent was obtained from the parents of the patient.

## Discussion and conclusions

In this study, we described clinical and molecular findings in a female with mild speech and motor developmental delay, short stature, recurrent pulmonary infections, tooth agenesis and triad of BLTS. Using CNV-seq, we identified a 3.1 Mb de novo interstitial deletion of the 14q13.2q21.1 region encompassing 17 OMIM genes.

The prevalence of chromosome 14q11-q22 deletion syndrome is lower than 1 in 1,000,000 infancies. The phenotype of patients is largely determined by the deletion size and breakpoints. Several candidate genes were well identified like *FOXG1*, *NKX2*–1 and *PAX9*. Deletions including *FOXG1* and a 1 Mb upstream region on 14q12 cause severe intellectual disability [5, 12–16]. *NKX2*–1 (14q13.3) deletion is responsible for choreoathetosis, hypothyroidism, short stature and neonatal respiratory distress and haploinsufficiency of *PAX9* (14q13.3) causes oligodontia phenotype [5, 17–21]. The role of other genes is still under evaluation. *NPAS3* (14q13.1) encodes a transcription factor localized to the nucleus and may regulate genes involved in neurogenesis. Npas3^−/−^ mice had abnormal neurodevelopment, neurosignaling and behavior [22], making it a candidate gene of holoprosencephaly (HPE) and hypoplasia of the corpus callosum (ACC) in 14q11-q22 deletion syndrome patients [5, 23]. *RALGAPA1* (14q13.2) encodes a major subunit of the RAL-GTPase activating protein, and was suggested to be important in brain development [24]. *NFKBIA* (14q13.2) mutation cause dominant inherited ectodermal dysplasia and immunodeficiency 2 (EDAID2, OMIM 612132) and may be the explanations of patients’ immunological features [4, 8]. *SEC23A* (14q21.1) encodes a subunit of a protein complex and found in the ribosome-free transitional face of the endoplasmic reticulum and associated vesicle and it is considered as a candidate gene of joint hyperlaxity [8, 17]. Mice with knock-out had abnormal cartilage development and collagen level [25]. Biallelic *SEC23A* mutation causes craniolenticulosutural dysplasia (OMIM 607812). It is characterized by facial dysmorphism, late-closing fontanels, cataract, and skeletal defects including joint laxity [26].

**Fig. 3:**
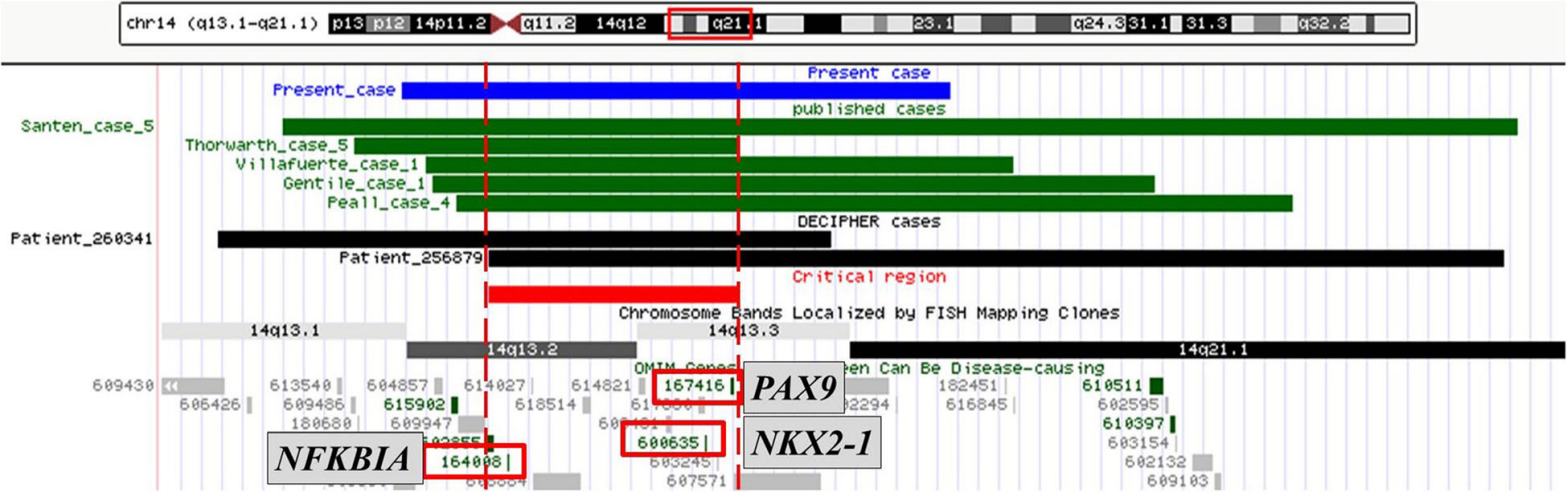
Schematic representation of the chromosomal region deleted of our patient (blue) and other similar deletions at 14q13.2-q21 described in the previously reported literature (green) and DECIPHER database (black). Three critical genes were located in the overlapping region

The deletion region of our patient (chr14: 35,268,524–38,367,321) encompasses *RALGAPA1* and *NFKBIA* but no *NPAS3* or *SEC23A*. Consequently, joint laxity, HPE or ACC was not observed in our patient. Epilepsy was also absent in our patient even though *RALGAPA1* was deleted. We reviewed published ten patients with phenotype description and similar deletion regions encompassing *RALGAPA1*, the deleted sizes ranged from 0.82 Mb to 6.98 Mb, and only one patient with 1.99 Mb deletion reported by Caliebe A et al. had seizures [4, 5, 8, 19, 23, 27–29]. Therefore, deletion of *RALGAPA1* was not sufficient to cause seizures and the genotype-phenotype correlation of *RALGAPA1* deletion remained unclear. Considering our patient had decreased IgG, paranasal sinusitis and recurrent infections, we also reviewed immunological features of seven patients with *NFKBIA* deletion (two patients were collected from DECIPHER database, Fig. 3). Patient reported by Villafuerte B et al. also had low IgG but also relatively low lymphocyte count and percentage of switched B cells [8]. Gentile M patient also had recurrent infections, with a mild reduction of CD3/CD8 lymphocytes and an elevation of CD4/CD8 ratio, yet her IgA, IgG, and IgM were normal [4]. Santen G patient 5 and Peall K patient 4 had recurrent lower respiratory infections [5, 19]. Patient 256,879 from DECIPHER database also had recurrent infections. We next reviewed seven patients with *PAX9*, *NKX2–1* deletion and leaving *NFKBIA* intact in previously literature. The deleted sizes ranged from 0.36 Mb to 3.69 Mb, and only one male with 2.34 Mb had recurrent bronchitises [5, 17, 19, 20, 29]. It was notable that the pLI (probability of LoF intolerant) value of *NFKBIA* was 1, and the o/e score is 0 (0–0.19), indicating deletion of this gene may have serious clinical consequences. In addition, three *NFKBIA* nonsense variants were reported in patients with EDAID2 [30]. To this respect, *NFKBIA* haploinsufficiency may be an appropriate explanation of the immunological features of 14q13 deletion patients.

Our patient, together with previously reported patients, identified a well-defined, more benign, 14q13 distal microdeletion syndrome. The major phenotype includes choreoathetosis, tooth agenesis, pulmonary dysfunction, immunological abnormal and hypothyroidism. The 1.4 Mb critical region contained at least three candidate genes: *NFKBIA*, *NKX2*–1 and *PAX9* (Fig. 3). In this region, one more gene, *PSMA6*, with its pLI being 1 and o/e score being 0 (0–0.23), should be more attention. *PSMA6* encodes the component of the 20S core proteasome complex involved in the proteolytic degradation of most intracellular proteins. Variants in *PSMA6* were reported to be associated with inflammation diseases like myocardial infarction [31], arthritis [32, 33], etc. Given its essential function and intolerant of LoF variants in population, more subtle features may be uncovered in the future.

Previous researches have demonstrated that CNV occurred in 5–10% of the total human genome [34], and chromosomal microarray analysis (CMA), as a stable and accurate platform, is used for detecting. Currently, CNV-seq is developed by analyzing data generated from WES or whole genome sequencing (WGS) [35–37]. Recent studies showed that the combination of WES and CNV-seq by low cover genome sequencing increased diagnostic yield in patients with rare diseases [38, 39]. In our study, after suspecting the diagnosis of BLTS of our patient, we performed trio-WES and low coverage WGS (0.3X) simultaneously. No putative pathogenic variants in *NKX2*–1 was identified, but we uncovered a 3.1 Mb deletion encompassing *NKX2–1*, as well as *PAX9* and *NFKBIA.* Notably, WES data could be applied to CNV identification, so we used WES data to validate this deletion. By reviewing our deleted region and other reported patients, we proposed a 1.4 Mb critical region for 14q13 distal microdeletion syndrome with a well-defined, more benign phenotype compared to 14q11-q22 deletion syndrome. Our results further demonstrated the clinical utility of the diagnostic strategy combining WES and CNV-seq for genetic diseases patients.

## Data Availability

The datasets used and/or analyzed during the current study are available from the corresponding author on reasonable request.

## Abbreviations

ACC: Hypoplasia of the corpus callosum
BLTS: Brain-lung-thyroid syndrome
CMA: Chromosomal microarray analysis
CNVs: Copy number variations
CNV-seq: Copy number variation sequencing
CT: Computed tomography
HPE: Holoprosencephaly
pLI: Probability of LoF intolerant
SNVs: Single nucleotide variants
WES: Whole exome sequencing
WGS: Whole genome sequencing
